# Academic emotions and attitudes regarding interprofessional collaboration in health care activities: a prospective study among newly arrived physicians participating in a fourteen-week course

**DOI:** 10.1186/s12909-023-04620-7

**Published:** 2023-09-07

**Authors:** Hanna Lachmann, Caroline Löfvenmark

**Affiliations:** 1 Department of Learning, Informatics, Management and Ethics, Karolinska Institutet, 171 77 Stockholm, Sweden; 2 Department of health promoting science, Sophiahemmet University, P. O. Box 5605, 114 86 Stockholm, Sweden

**Keywords:** Newly arrived physicians, Academic emotions, Flow, Stress, Interdisciplinary Education Perception Scale (IEPS), Interprofessional collaboration, Contextual Activity Sampling System methodology (CASS)

## Abstract

**Background:**

One way of facilitating entrance into the Swedish health care system, for newly arrived physicians from outside the European Union/European Economic Area, could be to set up and offer a course aimed to enhance understanding of it. This course was offered to increase insight about clinical practices, interprofessional teamwork and topics such as, Swedish health care laws, culture, and ethics. Acceptance of, and a flexible attitude towards, interprofessional teamwork are important for maintaining both the physician’s professional identity and a high quality of patient care.

The aim of this study was to investigate newly arrived physicians’, academic emotions, experience of stress and flow during a fourteen-week course, as well as attitudes to interprofessional collaboration, both before and after.

**Method:**

A prospective study was conducted, with participants asked to respond on one questionnaire every course day, by using the Contextual Activity Sampling System methodology. The participants were asked to complete a questionnaire comprising ten questions about ongoing activity and in what way they experience, e.g., collaboration, interprofessional teamwork, academic emotions, flow, and stress. Furthermore, the participants were asked to score their attitude towards interprofessional teamwork by using the interdisciplinary education perception scale both before and after the course.

**Results:**

The total sample comprised 27 qualified physicians, from outside the European Union/European Economic Area. In the interdisciplinary education perception scale category, “perception of actual cooperation¨, the participants had significantly higher scores after the course. Flow and academic emotions were felt mostly during own periods of study, seminars, and lectures. The academic emotions were apathy, anxiety, and boredom. The most frequently experienced academic emotion was apathy. Course participants rated stress highest in connection with the examination.

**Conclusion:**

The results show that the course had a positive impact on the participants perception of actual cooperation. It appears that participating in this kind of course was a positive experience for the participants, since they mostly experienced high levels of flow. Collaborating with others was experienced as positive, with participants reporting a high degree of flow in activities during collaboration.

## Background

In recent years, increasing numbers of people from outside the European Union (EU)/European Economic Area (EEA) have applied for residence permits to live and work in Sweden. Some are well-educated, medically qualified persons, e.g., physicians. Concerning the Swedish National board of Health and Welfare (2023) [1] it takes about 2—4 years for physicians from outside EU/EEA to get their registration approved for possibility to work as a physician in Sweden (https://www.socialstyrelsen.se/en/apply-and-register/doctors-specialist-medical-training/) The physicians may meanwhile encounter demands about the possibility to start working in their profession in another country [2]. Demands may concern the need of knowledge about, and applying for, recertification. This depends on whether their education satisfies the requirements of the current Swedish health care system [1–3]. The need to understand both the Swedish language and context is sometimes underestimated, thus causing problems for a smooth professional transition. Successful integration requires an understanding of both language and cultural differences in a healthcare context. Also, physicians from outside the EU/EEA need to learn about, and understand, the Swedish context regarding cultural, theoretical, ethical, and practical knowledge [4].

Learning is not only about cognitive activity but also involves so-called academic emotions, such as stress, anxiety, boredom, enthusiasm, and joy [5]. This means that study and learning establish a multifaceted interplay concerning engagement, performance and emotions connected to experienced self-appraisal [6]. Accordingly, Pekrun et al. [5], Adar [6] and Ekornes [7] highlighted learning as strategies including creative and flexible pedagogy, stimulating positive academic emotions. Furthermore, to be able to manage progress during learning situations tasks has to be challenging for give possibility to make progress. When a task experience as satisfactory concerning the level of both competence and challenge a situation optimal for learning described as flow occurs, while, e.g., low competence in combination in combination with high challenge might give feeling of anxiety [8–11].

To maintain the professional identity of physicians, from outside the EU/EEA, acceptance and flexibility concerning interprofessional teamwork (IPT) have shown to be important [12–14]. IPT is defined as different healthcare professions, patients, their relatives, and other non-clinician's working together to enable best possible quality of care [15]. IPT is often an overlooked factor that must be a priority for each professional person’s contribution to patients’ care [16, 17]. Also, the World Health Organization (WHO) [18] emphasized the importance of IPT regarding patient safety. The Interdisciplinary Education Perception Scale (IEPS) [19] is a reliable and valid questionary used to find out respondents self-estimated attitude concerning IPT.

The importance of lifelong learning, teamwork and empathy were emphasized in a study by San-Martín et al. [20] and the impact on physicians’ stress, health and professionalism has been highlighted by Delgado-Bolton et al. [21]. Wolanik et al. [22] highlighted the importance of language and socially acting in a correct way as important for interaction regarding foreign doctors’ professionalism. Expectations regarding the physicians’ professional role in Sweden include carrying out their work competently and being responsible for making medical decisions despite predictable challenges [23].

One way to facilitate and support physicians from outside the EU/EEA might be to facilitate their entrance to the Swedish health care system, by setting up and offering a course focussing on the Swedish context.

Schot et al., [24] stressed that different health care professions have their own culture involving, e.g., beliefs, values, attitudes, and performances that both contribute to, and may be challenging for, IPT. Also, Best et al. [16] emphasize the importance of understanding the roles of other professions roles in an interprofessional team.

Academic emotions, such as stress regarding different learning activities during the ongoing course were of interest to investigate in that the academic context should not be seen as simply comprising cognitive activities. Both Pekrun et al., [5] and Adar [6]. have in their research pointed out that so-called “academic emotions”, e.g., stress, boredom, anxiety, joy, and enthusiasm constitute a complex interplay between engagement, performance and emotions tied to the individual’s self-appraisal.

Compared to outside EU/EEA country’s the Swedish health care system could be understood as different for newly arrived physicians [1]. To find out needs and processes that could facilitates their admission process for working in our healthcare system we investigate their experience among participants during this course.

## Method

### Aim

The aim of this study was to investigate newly arrived physicians’, academic emotions, experience of stress and flow during the course, as well as attitudes to interprofessional collaboration, both before and after.

### Design

A prospective study was conducted during a fourteen-week course given to newly arrived physicians.

In this study, the participants reported their self-rated perception and development during various activities by using the Contextual Activity Sampling System (CASS) methodology [25–27]. The CASS-methodology was developed for investigating frequent data during ongoing activities, about how, e.g., emotional experiences impact on motivation concerning studies and the options for create new knowledge by using mobile data technology [28].

### Settings

This study took place at a Swedish university from August – December 2017. The course was voluntary, intended for physicians educated outside the EU and EEA and lasted for 14 weeks, aimed to facilitate entrance to the Swedish health care system. To be accepted for the course the applicants must have, a validated education according to Swedish regulations, applied for Swedish identification papers at the National Board of Health and Welfare [1]. During the application procedure, the participants were interviewed to assess their level of knowledge, if they understood and spoke Swedish sufficiently well, and if they had motivation and were able to complete course. The admission interviews were conducted by the course managers. The participants had not started work as physicians in Sweden when the course started.

All participants admitted (*n* = 36), were invited to participate in this study during their first day of the course. The participants were informed, both orally and in writing, about this studies intention and design, and that the results only were intended for research purposes. They were then asked if they were interested to participate and informed about that it was voluntary and that they may drop out whenever without consequences concerning participating the course.

The course focus was on the Swedish health care context to enhance the physicians’ understanding about the Swedish health care system, such as knowledge about regulations, clinical practices, interprofessional teamwork and other topics, e.g., Swedish health care laws, culture, and ethics. The course curriculum integrates both practical and theoretical components to enhance participants’ knowledge about healthcare regulations, relevant legislation and organisational structures, professional terms, interprofessional teamwork, care ethics, patient safety, and auscultation in various clinical workplace contexts. Furthermore, the participants were offered clinical training in different health care context and at a clinical training centre with special trained tutors. This included realistic simulation scenarios involving evidence-based methods, both acute and non-medical, and surgical situations focussing on interprofessional team communication. The participants were scheduled to attend three days a week at campus for seminars, at the clinical training centre or at the hospital for auscultation, and two days a week were allotted to own studies.

The course was initiated in collaboration between the Swedish government and one Swedish university to facilitate a faster approved registration for the participants to be able to work as physicians.

## Data collection

### Participants

The total sample finally consisted of 27 educated physicians, 14 females and 13 males. The mean age of the responded participants was 35 years. Demographic data, such as age, gender, number of years in the profession before arriving to Sweden were collected on the first day of the course. Also collected were data, such as degrees other than a medical degree and experience of interprofessional education (IPE). IPE defined as, ‘occurs when two or more professions learn about, from and with each other to enable effective collaboration and improve health outcomes’ [13, 17, 18, 29].

## Data collection

The participants answered questionnaires that were distributed daily (*n* = 66), to all participants, during the fourteen weeks course. A total of 2,376 questionnaires were distributed via mobile phone technology. In addition, the participants were asked to score their attitudes about interprofessional teamwork (IPT) by using the Interdisciplinary Education Perception scale (IEPS) questionaries before and after the course. In the IEPS questionaries interprofessional cooperation are used as IPT synonymous.

### The questionnaires

Every day's questionnaire consisted of ten questions, (Table 1) regarding ongoing activity and in what way the participants experienced, e.g., cooperation in the group of the course, academic emotions, flow, and stress. At the end of each day, Monday to Friday, the participants received a reminder in their mobile phones about complete their daily CASS questionnaire.

**Table 1:** Daily CASS questions asked to be answered related to reported learning activities

**Positive emotions**							
1. Interested	7	6	5	4	3	2	1
2. Enthusiastic	7	6	5	4	3	2	1
3. Determined	7	6	5	4	3	2	1
**Negative emotions**							
4. Irritated	7	6	5	4	3	2	1
5. Nervous	7	6	5	4	3	2	1
6. Afraid	7	6	5	4	3	2	1
7. **Stress**	7	6	5	4	3	2	1
8. **Importance**	7	6	5	4	3	2	1
9. **Commitment**	7	6	5	4	3	2	1
10. **Flow**							
Challenge	7	6	5	4	3	2	1
Competence	7	6	5	4	3	2	1

Please indicate the degree to which you agree or disagree with the number of the response that the best expresses your feeling. The scale is as follows: 7. strongly agree, 6. agree,5. somewhat agree, 4. neither agree or disagree, 3. somewhat disagree, 2. disagree, 1. strongly disagree

The CASS methodology enables moment by moment reports from the respondents. The methodology was developed to gather on-going experiences instead retrospective memories [27]. The CASS-methodology was inspired by the Experience-Sampling Method (ESM) [30]. In this study the participants were asked to respond on one CASS-questionnaire each day during the course. The number of questionnaires completed ranged from zero to 58.

To be included in the analysis, the participants had to respond to the two provided IEPS questionnaires, before and after the course. In addition, the participants had to have completed a minimum of three CASS-questionnaires during the 14-week course. Nine of the participants completed less than three questionnaires and thus were excluded from the analysis. This resulted in the inclusion of 27 participants whereof 24 also answered the requested IEPS-questionnaires, before and after the course.

Procedure using Contextual Activity Sampling System.

By using the the Contextual Activity Sampling System (CASS) methodology, [25–27], for data collection the participants accessed the CASS questionnaires online. Each participant was asked to respond to a total of 66 questionnaires via CASS, each including ten questions (Table 1). The response times were 3 – 5 min for each questionnaire and the questions were repeated daily so they would become familiar to the participants. The questionnaires were returned to the database server automatically after completion. Data were stored and then made available to the group of researchers. This methodology enabled investigators to follow participants’ ongoing activities and experiences concerning, e.g., academic emotions, longitudinally. The focus was on collecting the participants’ experiences of collaboration, academic emotions, flow, and stress during a fourteen-week course, by using the CASS methodology. The study participants, physicians from countries outside Europe, were asked to work together in various activities, such as seminars, clinical practice and groupwork with, and without, supervision.

### Flow

**Fig. 1:**
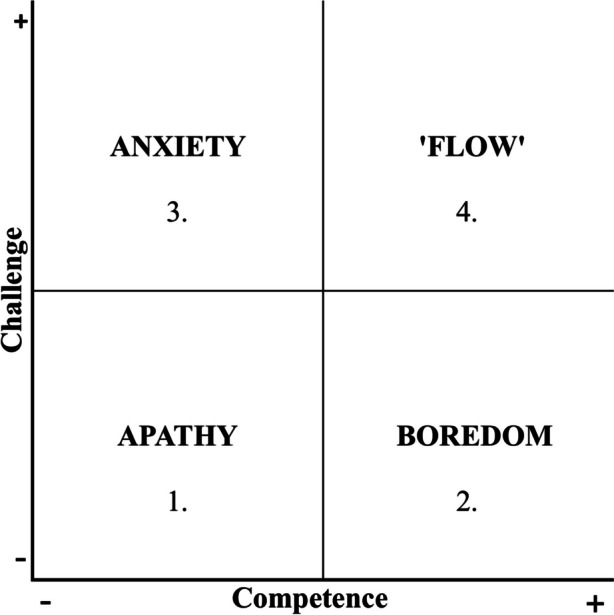
The four-channel model of flow inspired by Csikszentmihalyi [8]

The four-channel model [8, 10, 11] (Fig. 1) was used to describe the participants’ experience of flow in relation to ongoing activities.

Flow has been defined as a situation when a person carrying out a task experiences it as challenging, meaningful and has a feeling of having adequate competence to manage it [8–11]. Pekrun et al. [5], and Adar [6] have both stated that positive experiences stimulate people and help them appreciate the actual situation.

### Academic emotions

The participants rated their experience of academic emotions connected to daily course activities on a Likert scale graded from one to seven. One indicates very low and seven very much. The positive emotions rated were: determination, enthusiasm, and interest, and the negative emotions: irritation, nervousness, and being afraid. The participants were asked to rate their experience of both positive (determination, enthusiasm, and interest) and negative emotions (irritation, nervousness, and fear), together known as academic emotions [31]. The Cronbach´s alpha coefficient for the questions about positive emotions were 0.78 and negative emotions 0.79 [32].

### Stress

Experience of stress was measured by one-single question [33], on a Lickert-scale 1–7, with 1 indicating “Not at all” and 7 “maximum”. The question asked was: Stress refers to situations in which people feel tense, anxious, nervous, or crowded, or when they have sleeping difficulties because they are constantly thinking of things. Do you feel that kind of stress currently. The single-item measure of stress symptoms has been tested for validity by Elo et al. [33] and has been found to be satisfactory.

### Interdisciplinary Education Perception Scale

Using the Swedish version [34] of the Interdisciplinary Education Perception Scale (IEPS) [19], the participants’ attitudes and perceptions concerning the need of interprofessional teamwork were assessed. The IEPS [19, 34] consists of 12 questions with a six-point, Likert scale (one = strongly disagree and six = strongly agree) and was used to investigate if the course impacted on their perceptions. It has been divided into three categories, which are Competency and autonomy (scale 1–30), Perceived need for cooperation (scale 1–12), and Perception of actual cooperation (scale 1–30). The Cronbach´s alpha coefficient for the subscales were 0.89, 0.88 and 0.66 [19, 34].

### Analysis

Descriptive statistics were used to analyse demographic data, academic emotions, stress and FLOW. Group data are presented as mean value (± SD), numbers, and proportions of numbers (%). Since each participant answered the CASS-questions, i.e., stress, experience of flow, academic emotions, positive and negative emotions, between 3 – 58 times, a Z-score was calculated on an individual level: first, the mean and standard deviation for each question and each participant. The scores were then standardized for each question and for each participant by setting the mean to zero and the SD to one to reduce effects of variances related to individual answering tendencies [35]. Flow, apathy, anxiety, and boredom are presented with numbers and proportion of numbers in relation with learning activities. The experience of flow occurs when competence is rated > 0 and challenge > 0; anxiety when competence is rated < 0 and challenge > 0; apathy when competence is rated < 0 and challenge < 0; and boredom when competence is rated > 0 and challenge < 0 [8–10, 36]. The relation between stress and reported activities was also calculated, as well as positive and negative emotions connected to reported learning activities with descriptive statistics. IEPS was analysed by paired sample T-test.

All statistical analyses were performed using a database application (Excel) and the software The Statistical Package for Social Science, SPSS version 27 [37]. A *p*-value < 0.05 was considered as statistical significance.

## Results

### Demographic

Twenty-five of the participants reported how many years they had worked in their profession before arriving in Sweden. The mean of years in the profession was 5.4 years. Twelve of the participants had been working in another job in Sweden prior to the course, such as assistant nurse, researcher, personal assistant, salesman and teacher. Some of the participants reported having had two other jobs. Nine had a university degree other than their medical degree. Nine had previous experience of IPE, in which students from diverse professions studied/worked together with a mutual aim. No significant gender differences were identified. (Table 2). Please move Table 2 from above and move it to below this paragraph.

**Table 2:** Characteristics of the participants in the study

Variable	Total	Men	Women
	*n* = 27	*n* = 13	*n* = 14
Age, years mean (± SD)	35.2 (± 6.7)	35.4 (± 6.4)	35.1 (± 7.2)
Range, years	27–51	27–47	28–51
Years in profession before Sweden years (± SD) *n* = 25	5.4 (± 5.5)	5.4 (± 5.9)	5.4(± 5.5)
Working in another profession *n* (%) *n* = 26	12 (44.4%)	5 (38.5%)	7 (50%)
Another university degree *n* (%) *n* = 26	9 (34.6%)	5 (38.5%)	4 (28.6%)
Experience of IPE n (%) *n* = 27	9 (33.3%)	3 (23.1%)	6 (42.9%)

Demographic data was analysed by descriptive statistics. Data are presented as mean value (± SD), numbers, and proportions of numbers (%)

### Interdisciplinary education perception scale

Of the 27 participants, 22 (81%) completed the IEPS-questionnaire both before and after the course, except for the category ‘Perceived need for cooperation’, where 23 participants filled out both questionnaires. In the category, ‘Perception of actual cooperation’, the participants rated significantly higher scores after the course (23.9 SD ± 3.5 vs. 25.6 SD ± 2.7, *p* = 0.008). However, there was no change in rated answers before the course compared to after in the other two categories, ‘Competence and autonomy’ or ‘Perceived need for cooperation’ (Table 3).

**Table 3:** IEPS Before and after the course

Category (*n*)	Mean (± SD) before	Mean (± SD) after	*p*-value
Competency and autonomy (22)	24.27 (± 2.0)	24.91 (± 2.7)	0.204
Perceived need for cooperation (22)	10.41 (± 1.3)	10.50 (± 1.4)	0.780
Perception of actual cooperation (23)	23.87 (+ 3.5)	25.65 (+ 2.7)	0.008

IEPS was analysed by paired sample T-test. A *p*-value < 0.05 was considered as statistical significance

In the category, ‘Perception of actual cooperation’ the results showed a significant difference between women and men (women mean 25.1 SD + 2.8 vs. Men mean 22.1 + 3.6, *p* = 0.024) before the course started. This significant difference did not remain after the course (women mean 26.4 SD + 2.8 vs. Men mean 25.9 + 2.6, *p* = 0.234). The results did not show any significant differences in the other categories regarding gender.

In-group analysis for men and women over time showed a significant difference for women in the category, ‘Perception of actual cooperation’ (*p* = 0.008). The results did not show any significant differences in the other categories for women and there was no significant difference in any category for men.

### Questionaries answered via CASS

**Fig. 2:**
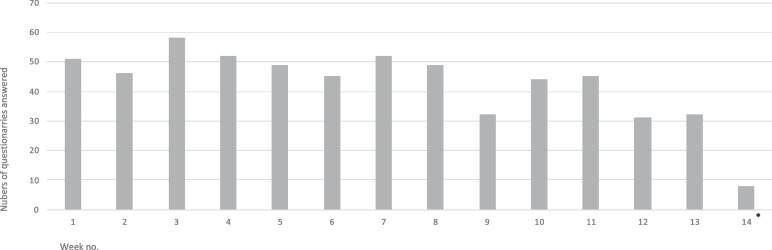
Diagram showing participants answered questionaries during the 14 week course. * During week 14 it was only two days scheduled for the participants

In a total 594 CASS-questionaries was answered during the course. The number of answered questionaries every week are shown in Fig. 2.

### Flow and academic emotions

The mean number of completed questionnaires was 23 (SD ± 16.27), maximum 58 and minimum four. The median was 18 questionnaires. The experiences of flow and academic emotions are shown in Table 4, related to reported learning activities. Most of the participants experienced a feeling of flow (*n* = 247) for all activities during the course. They felt it mostly during their own periods of study, seminars, and lectures. The most common academic emotion experienced by the participants was apathy (*n* = 140). This was experienced mostly during own periods of study and lectures. The second most common academic emotion was anxiety (*n* = 117).

**Table 4:** Experience of FLOW and academic emotions related to reported learning activities

Activity	FLOW *n* (%)	APATHY n (%)	ANXIETY n (%)	BOREDOM n (%)	TOTAL *n* (%)
Experience					
Own period of study	73(32.6)	75 (33.5)	36 (16.1)	40 (17.8)	224 (100)
Seminars	56 (43.1)	20 (15.4)	28 (21.5)	26 (20)	130 (100)
Lecture	45 (41.7)	24 (22.2)	18 (16.7)	21 (19.4)	108 (100)
Groupwork	31 (46.3)	7 (10.4)	15 (22.4)	14 (21.9)	67 (100)
Clinical training	11 (36.7)	1 (3.3)	12 (40)	6 (20)	30 (100)
Clinical practice	19 (70.4)	2 (7.4)	4 (14.8)	2 (7.4)	27 (100)
Other	4 (21)	9 (47.4)	3 (15.8)	3 (15.8)	19 (100)
Examination	8 (66.7)	2 (16.7)	1 (8.3)	1 (8.3)	12 (100)
Total (*n*)	247	140	117	113	

Flow and academic emotions are presented with numbers and proportion of numbers (%) in relation with learning activities

### Stress

**Fig. 3:**
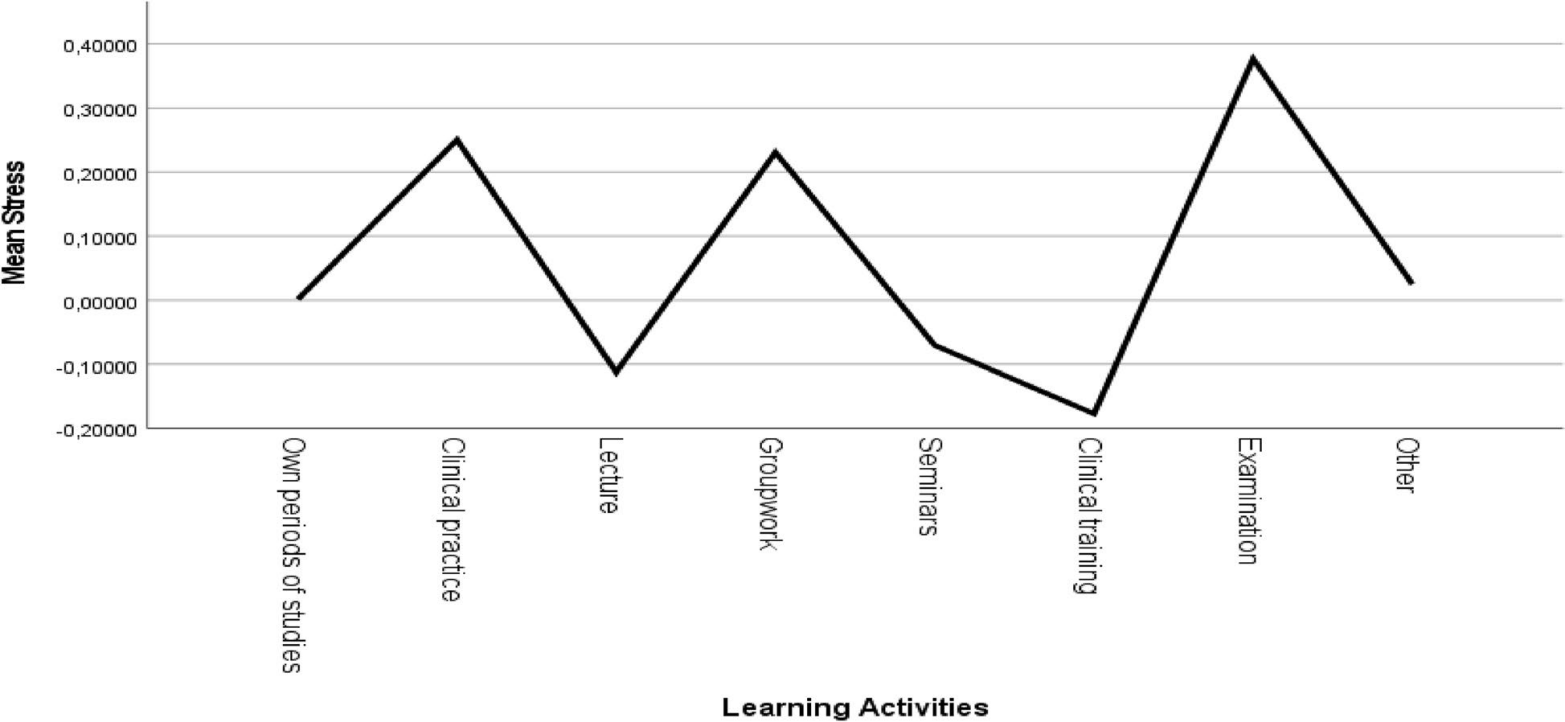
Z-score of stress connected to different learning activities. The highest stress was experienced during examination

The participants were also asked to rate their stress level, on a Likert scale 1–7, (7 indicate high stress) connected to reported activities; in total the participants answered the question 592 times. Regarding stress, the course participants rated stress at its highest connected to examination. They also gave high stress ratings during two learning activities, namely clinical practice and groupwork. The lowest rated stress was during clinical training (Fig. 3).

### Positive emotions connected to reported learning activities

**Fig. 4:**
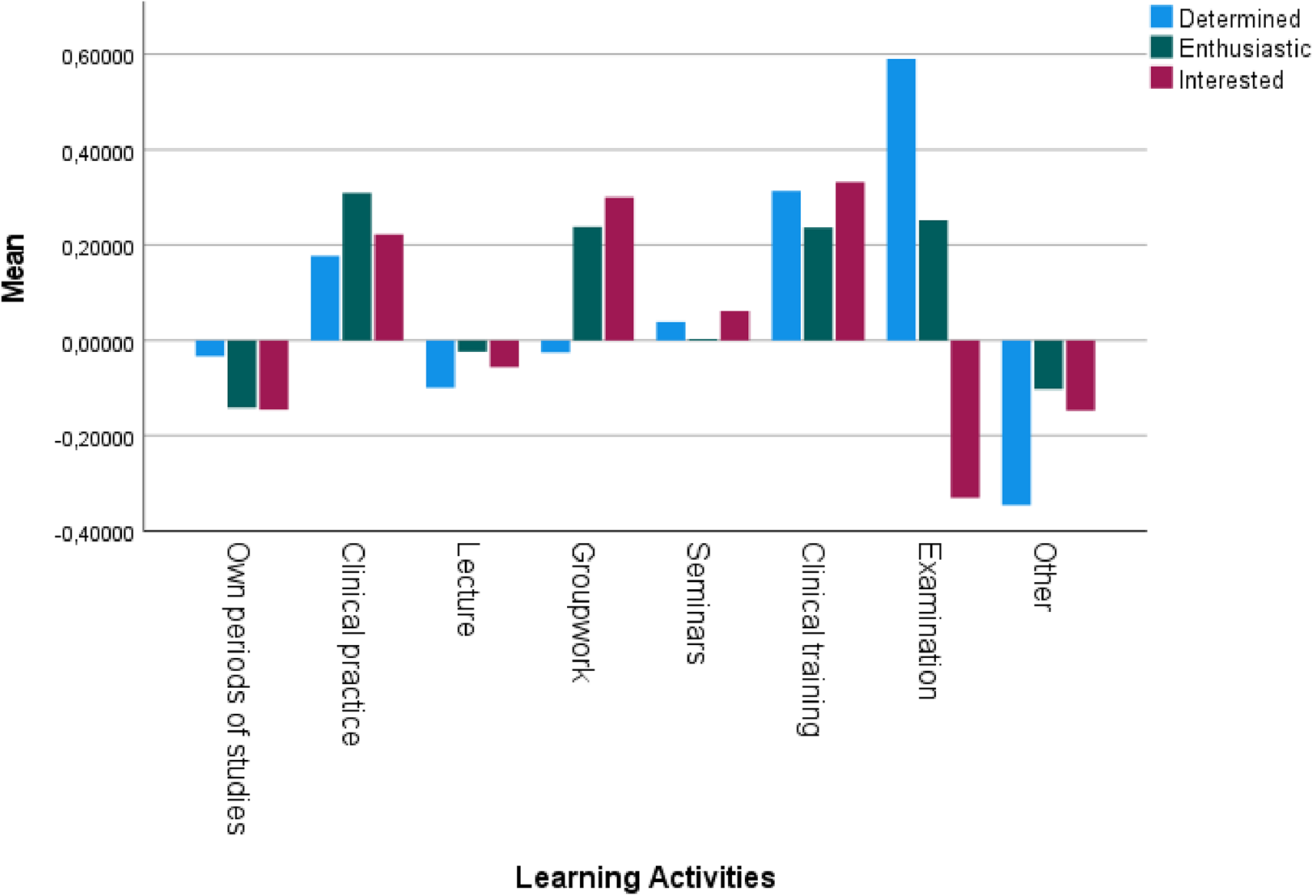
Z-scores mean of perceived rated positive emotions: determination, enthusiasm, and interest regarding different learning activities. Others = discussions, evaluation of the course, no studies, graduation ceremony

The participants' perceptions concerning positive emotions during examinations were highest regarding the determination and lowest for interest. They experienced enthusiasm during learning activities, such as clinical practice, clinical training, groupwork and examinations. Most interest was reported during clinical training, groupwork and clinical practice (Fig. 4).

### Negative emotions connected to reported learning activities

**Fig. 5:**
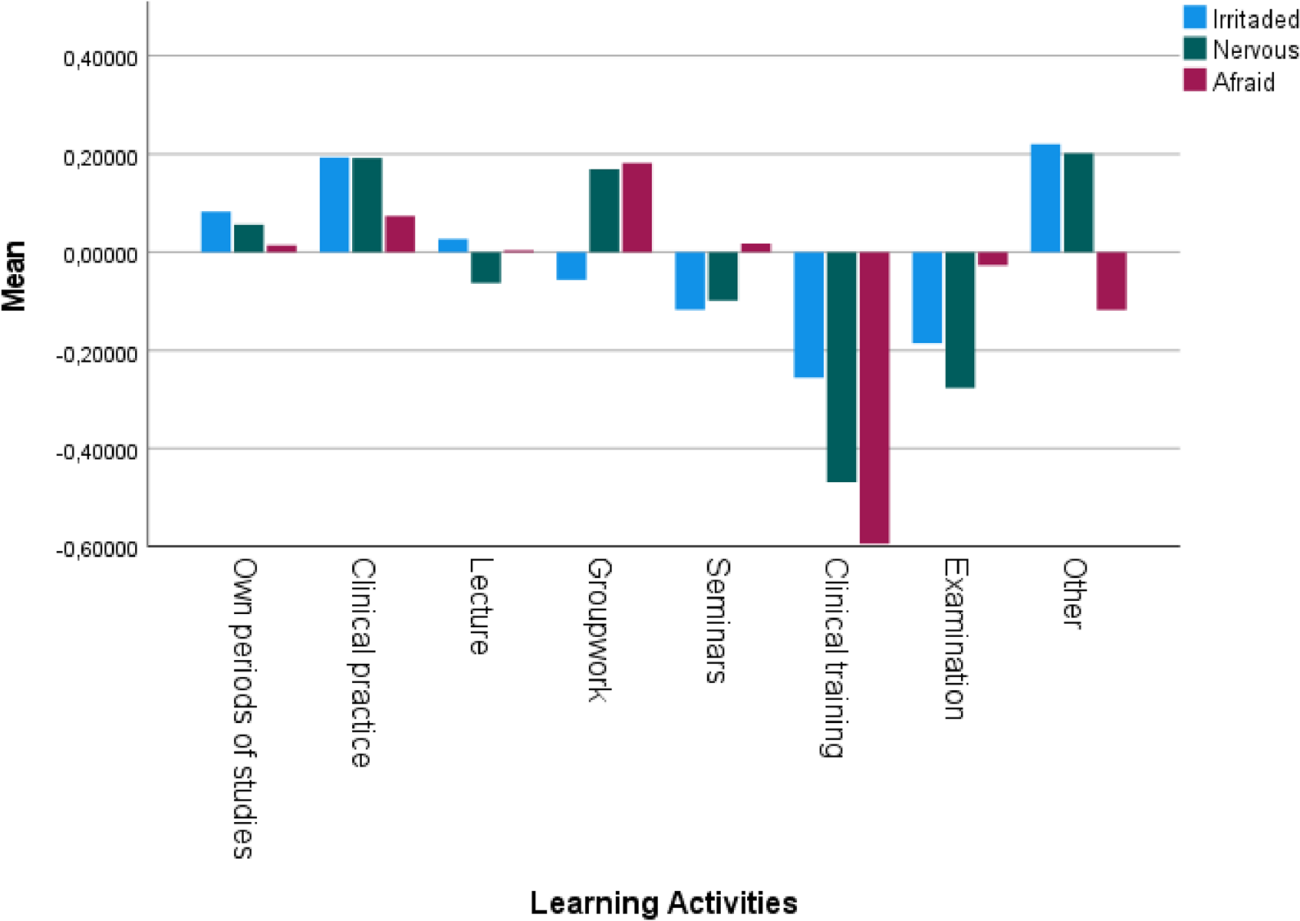
Z-scores mean of perceived rated negative emotions: irritated, nervous, and afraid regarding different learning activities. Others = discussions, evaluation of the course, no studies, graduation ceremony

The course participants gave high ratings for negative academic emotions, such as being afraid, nervous, and irritated, during clinical practice. High ratings were also found regarding being afraid and nervous during groupwork. Similarly, participants also gave high ratings for negative emotions, such as being irritated and nervous in the activities referred to as ‘Others’ (Fig. 5).

### Collaboration during different learning activities

When engaged in learning activities, the participants reported that collaboration occurred in 255 (56%) of 455 reported activities. Collaboration took place during seminars *n* = 74 (29%), own period of study *n* = 57 (22.3%), lectures *n* = 58 (22.7%), groupwork *n* = 35 (13.7%), clinical training *n* = 13 (5%) and clinical practice *n* = 9 (3.5%).

## Discussion

Thus, the aim of this study was to investigate newly arrived physicians’, academic emotions, experience of stress and flow during a fourteen-week course as well as their attitudes to interprofessional collaboration, both before and after. The participants’ perception and development during various activities were observed using the CASS methodology. In previous studies [38] it has been shown that using CASS improves systematic reflection, something that may impact on the participants' attitudes to, and experiences of, interprofessional teamwork.

The results showed significant differences in the IEPS in the category, ‘Perception of actual cooperation’, both for the whole group and in the group of female participants. This may indicate that women have a more positive attitude to teamwork. This result is in line with previous studies [39, 40] showing that female students are more positive to teamwork. In the study by Wilhelmsson et al. [39], medical and nursing students were included. The Readiness for Interprofessional Learning Scale (RIPLS) was used, and the results showed female students to be more positive to teamwork, regardless of educational programme. D'Costa et al. [41] also highlighted differences among women and men concerning attitudes to being educated as well as to engage with other disciplines. The significant results in the category, ‘Perception of actual cooperation’ are important in that they may be due to the participants being offered clinical training during this course. The opportunity for clinical training was helpful and aimed to enhance the development of the Swedish health care team culture by a sense of belonging as a team member. Why the other characteristics ‘Competence and autonomy’ and ‘Perceived need for cooperation’ remained unchanged during the course may depend on whether they were highlighted or discussed in the same way.

The results have also shown that the participants experienced high levels of flow while they participated in both seminars and lecturers, while the rated feeling of apathy and flow was similar. However, stress was low during own studies. This might depend on difficulties in finding out what exactly to do during time for own studies. Furthermore, that the participants experienced high levels of stress associated with examinations, clinical training and groupwork may be due to verbal and linguistic barriers. It is interesting that positive emotions during examinations were rated as high regarding determination, and low regarding interest. Also, that enthusiasm was experienced during clinical practice, clinical training, groupwork and examinations. In a study by Järvenoja et al. [42] that investigated situation-specific challenges in higher education, the results showed that students felt positive emotions when being challenged during collaborative group work. Also, that perceived interest during clinical training, groupwork and clinical practice was reported as positive. We may consider situations like examination and clinical training to be challenging. It was surprising that clinical practice was experienced as irritating, and the learning activities of clinical practice and groupwork evoked feelings of nervousness and being afraid. Pekrun et al. [43] suggested that the complexity of academic emotions also depends on the relation between the person's condition, experienced emotions, and the activity’s level of demands.

Although there was a small group in this study, we can see that they acknowledged the importance of teamwork. The World Health Organization [44] has identified teamwork as being important in health care. The results also show that ‘Perception of actual cooperation’ was higher after the course, which indicates that the participants discovered the importance and benefits of team cooperation during the course.

### Limitations and strengths

In total, the participants, received 66 daily questionnaires regarding flow and academic emotions, connected to activities during the course. The mean of completed questionnaires was 23, but the data collected were enough to draw the conclusions presented below. Some misunderstandings have been identified in the participants' written answers in the questionnaires that may be because of linguistic difficulties when reading Swedish. Two strengths with this study are that it includes both women and men, with different experiences of working as physicians, and that the participants were followed every day of the course.

## Conclusion

The results in present study showed that the course had a positive impact on the participants perception of actual cooperation. Participating in the course appeared to be a positive experience for the participants, since they mostly rated flow for its duration. Collaborating with others was experienced as positive, as the participants rated high levels of flow in activities during collaboration. This type of course may help the participants to gain an insight into health care in Sweden. There is a need to study this further.

## Data Availability

The collected data and analyses were, and are, not accessible to the general public as they may include details about the participants. However, the data are available on request. Please contact the corresponding author.
